# Risikokommunikation bei der Eindämmung der COVID-19-Pandemie: Herausforderungen und Erfolg versprechende Ansätze

**DOI:** 10.1007/s00103-021-03283-3

**Published:** 2021-02-09

**Authors:** Julika Loss, Evgeniya Boklage, Susanne Jordan, Mirjam A. Jenny, Heide Weishaar, Charbel El Bcheraoui

**Affiliations:** 1 Abteilung für Epidemiologie und Gesundheitsmonitoring, Robert Koch-Institut, General-Pape-Str. 62–66, 12101 Berlin, Deutschland; 2 Zentrum für internationalen Gesundheitsschutz, Robert Koch-Institut, Berlin, Deutschland; 3 Projektgruppe Wissenschaftskommunikation, Robert Koch-Institut, Berlin, Deutschland; 4 Harding-Zentrum für Risikokompetenz, Universität Potsdam, Potsdam, Deutschland; 5 Zentrum für Adaptive Rationalität, Max-Planck-Institut für Bildungsforschung, Berlin, Deutschland

**Keywords:** Risikowahrnehmung, Risikokompetenz, SARS-CoV‑2, Krisenmanagement, Medien, Risk perception, Risk competence, SARS-CoV‑2, Crisis management, Media

## Abstract

Risikokommunikation spielt eine zentrale Rolle in Public-Health-Notlagen: Sie muss informierte Entscheidungen ermöglichen, schützendes bzw. lebenserhaltendes Verhalten fördern und das Vertrauen in öffentliche Institutionen bewahren. Zudem müssen Unsicherheiten über wissenschaftliche Erkenntnisse transparent benannt werden, irrationale Ängste und Gerüchte entkräftet werden. Risikokommunikation sollte die Bevölkerung partizipativ einbeziehen. Ihre Risikowahrnehmung und -kompetenz müssen kontinuierlich erfasst werden. In der aktuellen Pandemie der Coronavirus-Krankheit 2019 (COVID-19) ergeben sich spezifische Herausforderungen für die Risikokommunikation.

Der Wissensstand zu vielen wichtigen Aspekten, die COVID-19 betreffen, war und ist oftmals unsicher oder vorläufig, z. B. zu Übertragung, Symptomen, Langzeitfolgen und Immunität. Die Kommunikation ist durch wissenschaftliche Sprache sowie eine Vielzahl von Kennzahlen und Statistiken geprägt, was die Verständlichkeit erschweren kann. Neben offiziellen Mitteilungen und Einschätzungen von Expertinnen und Experten wird über COVID-19 in großem Umfang in sozialen Medien kommuniziert, dabei werden auch Fehlinformationen und Spekulationen verbreitet; diese „Infodemie“ erschwert die Risikokommunikation.

Nationale wie internationale Forschungsprojekte sollen helfen, die Risikokommunikation zu COVID-19 zielgruppenspezifischer und effektiver zu machen. Dazu gehören u. a. explorative Studien zum Umgang mit COVID-19-bezogenen Informationen, das COVID-19 Snapshot Monitoring (COSMO), ein regelmäßig durchgeführtes Onlinesurvey zu Risikowahrnehmung und Schutzverhalten sowie eine interdisziplinäre qualitative Studie, die die Konzeption, Umsetzung und Wirksamkeit von Risikokommunikationsstrategien vergleichend in 4 Ländern untersucht.

## Abstract (en)

Risk communication plays a central role in public health emergencies: it must enable informed decisions, promote protective or life-sustaining behaviour, and maintain trust in public institutions. In addition, uncertainties in knowledge must be named transparently; irrational fears and rumours must be refuted. Success factors for risk communication are the participation of citizens as well as the continuous recording of risk perception and risk competence in population groups. The current COVID-19 (corona virus disease 2019) pandemic poses specific challenges for risk communication.

The state of knowledge on many important aspects concerning COVID-19 was and is often uncertain or preliminary, e.g. on transmission, symptoms, long-term effects and immunity. Communication is characterised by scientific language and an array of figures and statistics, which can render the content difficult to understand. Alongside the official announcements and statements by experts, COVID-19 is widely communicated on social media, spreading misinformation and speculation; this “infodemic” can complicate risk communication.

Various national and international scientific projects will help tailor risk communication on COVID-19 to target groups and thereby render it more effective. These projects include explorative studies on how people deal with COVID-19-related information; the COVID-19 Snapshot Monitoring (COSMO) project, a regularly conducted online survey on risk perception and protective behaviour; and an interdisciplinary qualitative study that compares the design, implementation and effectiveness of risk communication strategies in four countries.

## Einleitung

Die weltweite Pandemie der durch das Virus SARS-CoV‑2 (severe acute respiratory syndrome coronavirus type 2) ausgelösten Erkrankung COVID-19 hat die erhebliche Relevanz der Risikokommunikation hervorgehoben. Da die Eindämmung des Infektionsgeschehens wesentlich vom präventiven Verhalten der Gesamtbevölkerung abhängt (u. a. Tragen einer Alltagsmaske, Wahren von physischem Abstand), müssen die Menschen über dieses Schutzverhalten sowie dessen Notwendigkeit zeitnah, bedarfsgerecht und effektiv aufgeklärt werden. Der vorliegende Artikel wird zunächst auf die Rolle von Risikokommunikation bei Public-Health-Notlagen allgemeiner Art eingehen. Anschließend werden Kriterien erläutert, die für eine wirksame Risikokommunikation aufgestellt werden können. In der Folge werden spezifische Herausforderungen thematisiert, die die COVID-19-Pandemie an die Risikokommunikation stellt, und anhand aktueller internationaler Beispiele illustriert. Es werden 3 laufende Forschungsprojekte vorgestellt, die verschiedene Aspekte der Risikokommunikation zu COVID-19 untersuchen; daraufhin werden Empfehlungen für die Risikokommunikation bei COVID-19 abgeleitet. So soll der Artikel auch helfen zu verstehen, wie sich Wissenschaft, Public-Health-Praxis und Politik besser auf zukünftige Ausbruchsituationen und Krisen vorbereiten können.

## Risikokommunikation als zentrale Säule in der Bewältigung von Public-Health-Notlagen

### Was ist Risikokommunikation?

Eine zentrale Aufgabe von Public Health ist es, gesundheitliche Risiken zu verringern, die durch Lebensverhältnisse oder menschliches Verhalten entstehen, und gesundheitsförderliche Faktoren zu stärken. Die Kommunikation spielt dabei eine wichtige Rolle [1]. Gesundheitskommunikation umfasst die Vermittlung und den Austausch von Informationen, welche die Gesundheit und Gesunderhaltung, aber auch Krankheit, diagnostische und therapeutische Verfahren betreffen [2, 3]. Oft kommen hier Massenmedien über Funk, Fernsehen, Plakatwände und das Internet zum Einsatz [4, 5]. Als Teilbereich wird die Risikokommunikation abgegrenzt, die die Öffentlichkeit zielgerichtet über Risiken informieren soll, z. B. über die Art, Bedeutung und Kontrollierbarkeit eines Risikos [6]. Die Weltgesundheitsorganisation (WHO; [7]) definiert Risikokommunikation als „Austausch von Informationen, Empfehlungen und Meinungen zwischen Experten und der Bevölkerung angesichts von Bedrohungen für ihre Gesundheit und/oder ihr wirtschaftliches oder soziales Wohlergehen“. Lundgren und McMakin differenzieren darüber hinaus zwischen „Care Communication“, d. h. der Risikokommunikation, die sich auf riskante Lebensstile wie Tabakrauchen oder die Nebenwirkungen von klinischen Therapien bezieht [8, 9], und der „Crisis Communication“ (Krisenkommunikation) bzw. der Risikokommunikation in akuten Public-Health-Notlagen [10]. Sie kommt bei plötzlichen, unerwarteten Gefahren wie Pandemien, Naturkatastrophen, Hungersnöten oder Bioterrorismus zum Tragen.

### Warum ist Risikokommunikation in Public-Health-Notlagen wichtig?

Risikokommunikation gilt als zentrale Säule des Krisenmanagements bei Public-Health-Notlagen und ist entscheidend dafür, dass Maßnahmen zur Bewältigung der Krise erfolgreich umgesetzt werden [11]. Risikokommunikation muss informierte Entscheidungen ermöglichen, schützendes bzw. lebenserhaltendes Verhalten fördern und das Vertrauen in öffentliche Institutionen bewahren [12, 13]. Effektive Risikokommunikation kann die Risikokompetenz in der Bevölkerung fördern, also die Fähigkeit, informiert, kritisch und reflektiert mit Risiken umzugehen. Hierzu gehören statistisches Denken, heuristisches Denken, Systemwissen (z. B. über das Gesundheitswesen) und psychologisches Wissen [14].

Expertinnen und Experten bzw. Behörden müssen bei Public-Health-Notlagen schnell kommunizieren, um den Menschen ein Gefühl persönlicher Kontrolle wiederzugeben und Schaden abzuwenden; gleichzeitig müssen sie sich der Herausforderung stellen, dass in Krisensituationen die Sachlage oft unübersichtlich und unsicher ist und sich kontinuierlich ändert. In Public-Health-Krisen sind Bürgerinnen und Bürger meist akut besorgt über ihre Gesundheit und fragen daher gesundheitsbezogene und handlungsleitende Informationen verstärkt nach. Diese erhöhte Nachfrage, aber auch die Dynamik, mit der sich Sachlagen, Erfahrungen und Expertenwissen ändern können, führen zu einer hohen Frequenz an gesundheitsbezogenen Botschaften während Public-Health-Notlagen.

Zarocostas [15] zitiert eine WHO-Vertreterin mit der Aussage, dass jedes Ausbruchsgeschehen von einem „Tsunami an Informationen“ begleitet werde. Um unter diesen schwierigen Rahmenbedingungen Menschen zu erreichen und so informieren zu können, dass sie in der Lage sind, sich selbst und andere zu schützen – also risikokompetent handeln können –, muss Risikokommunikation bestimmte Kriterien erfüllen. Diese Kriterien basieren auf verschiedenen Modellen und Theorien für Risikokommunikation, wie sie unter anderem von Covello und Sandman [16] beschrieben wurden.

Die *Theorie des psychischen Lärms* (englisch „mental noise theory“) beispielsweise beschreibt, dass die Fähigkeit von Rezipientinnen und Rezipienten, Risikoinformationen wahrzunehmen und zu verarbeiten, stark beeinträchtigt ist, wenn sie unter Bedrohung oder Stress stehen [17]. Dieses Modell impliziert, dass Kommunikationsbotschaften während einer Krise klar und verständlich, gut strukturiert und leicht zugänglich sein müssen; Wiederholung und Visualisierung können die Klarheit der Kommunikation verbessern.

Die *Theorie der Vertrauensbestimmung* (englisch „trust determination theory“) geht davon aus, dass es für eine effektive Risikokommunikation unerlässlich ist, Vertrauen aufzubauen. Die größte Herausforderung besteht darin, dass die Öffentlichkeit in Krisenzeiten zunehmend skeptischer gegenüber Behörden und Autoritäten wird. Um glaubwürdig zu sein, muss die Kommunikation daher von Empathie, Kompetenz und Transparenz geprägt sein [18].

Das *Modell der negativen Dominanz* (englisch „negative dominance model“) basiert auf der Beobachtung, dass die Menschen in einer Krise, wenn sie verängstigt oder wütend sind, negativen Informationen und Ergebnissen mehr Aufmerksamkeit schenken und sich eher auf potenzielle Verluste denn auf mögliche Chancen und positive Entwicklungen konzentrieren [16]. Hinweise auf Gefahren sollten daher mit lösungsorientierten Botschaften flankiert werden.

Die *Theorie der Risikowahrnehmung* (englisch „theory of risk perception“) bezieht sich auf die Vorstellung, dass die Wahrnehmung von Gefahren oftmals durch Faktoren bestimmt wird, die nicht unbedingt mit dem tatsächlichen Schweregrad dieser Gefahren korrespondieren, sondern z. B. mit dem Grad an moralischer Empörung und gefühlter Hilflosigkeit. Diese sogenannten Empörungsfaktoren (englisch „outrage factors“ [19, 20]) zeichnen Chapman und Wutzke [21] am Beispiel der öffentlichen Reaktion auf geplante Mobilfunkmasten nach, wo z. B. unausweichliche und als unfair empfundene Risikoexposition, fehlende Kontrollierbarkeit sowie ausbleibende Dialogbereitschaft der Verantwortlichen zu einer großen öffentlichen Risikowahrnehmung führten. Als Gegenbeispiel eines gefährlichen Gesundheitsrisikos mit niedrigem Empörungsfaktor kann z. B. die Grippe (Influenza) gelten. Malecki et al. beschreiben, wie sich bei der COVID-19-Pandemie in den USA die Empörungsfaktoren über die Zeit verändern und damit die Risikowahrnehmung hinsichtlich SARS-CoV‑2 gestiegen ist [22].

Risikokommunikation muss auf die besonderen Eigenschaften der Risikowahrnehmung und Informationsverarbeitung in der Bevölkerung eingehen und auch verschiedene Bevölkerungsgruppen berücksichtigen, um in Public-Health-Notlagen handlungsleitende und lebensrettende Botschaften zu transportieren.

### Kriterien der Risikokommunikation in Public-Health-Notlagen

Kapazitäten und Kompetenzen zur Risikokommunikation sollten prophylaktisch und strategisch entwickelt werden und integraler Bestandteil von Bereitschafts- und Reaktionsplänen z. B. für Ausbrüche von Infektionskrankheiten sein. Die WHO hat einen Leitfaden veröffentlicht, um die am Krisenmanagement beteiligten Akteurinnen und Akteure dabei zu unterstützen, eine effektive Risikokommunikation zu implementieren [13]. Auch die US-amerikanischen Centers for Disease Control and Prevention haben ein Handbuch zur Krisen- und Notfallrisikokommunikation herausgegeben, das Theorie und Praxis der Reaktion auf Public-Health-Notlagen im öffentlichen Gesundheitswesen darstellt [23].

Aus den unterschiedlichen Modellen und Leitlinien zur Risikokommunikation lässt sich eine Reihe von Kriterien identifizieren, die in Public-Health-Notlagen berücksichtigt werden sollten.

#### Vertrauen und Glaubwürdigkeit.

In Public-Health-Notlagen müssen Menschen sehr schnell informiert und befähigt werden, sich selbst und andere zu schützen. Dies kann nur gelingen, wenn den Expertinnen und Experten bzw. Regierungsorganisationen, die über die Risiken aufklären und Handlungsempfehlungen geben, ausreichend Vertrauen entgegengebracht wird. Das ist nicht selbstverständlich, zumal gesundheitliche Krisensituationen Ängste schüren und die Skepsis gegenüber Behörden und Autoritäten erhöhen können [12, 24]. Die Art der Risikokommunikation kann dazu beitragen, Vertrauen in Botschaften und Institutionen herzustellen, z. B. indem fundierte, akkurate Informationen zeitnah, kompetent und verständlich vermittelt werden [13]. Die Botschaften müssen zudem empathisch sein und die betroffenen Bevölkerungsgruppen respekt- und verständnisvoll ansprechen [10, 24]. Es gibt auch Faktoren, die das Vertrauen der Bevölkerung in Risikokommunikation untergraben können, so zum Beispiel das Zurückhalten von Informationen, fehlende aktive Einbindung der Öffentlichkeit oder Uneinigkeit zwischen Expertinnen und Experten [10].

#### Eingeständnis von Unsicherheit.

In akuten Public-Health-Notlagen muss sich Risikokommunikation mit einer komplexen, sich ständig verändernden Ausgangslage und einem unvollständigen Wissensstand auseinandersetzen. Empfehlungen können sich immer wieder ändern, wenn die Krisensituation sich weiterentwickelt oder neue Erkenntnisse, z. B. zur Übertragung von Erregern, generiert werden [13]. Wenn Expertinnen und Experten oder Entscheidungstragende über Risiken informieren, sollten sie transparent machen, was bekannt ist, aber auch wozu die Kenntnisse bislang nicht ausreichen oder unsicher sind [25]. Botschaften sollten entsprechend formuliert werden [26, 27]. Gleichzeitig darf der wiederkehrende Hinweis auf die Unsicherheit des vermittelten Wissens nicht dazu führen, dass die Bevölkerung zusätzlich beunruhigt wird. Ziel von Risikokommunikation muss sein, dass sich Menschen innerhalb der Grenzen verfügbaren Wissens angemessen informiert fühlen [28].

#### Balance zwischen Alarmierung und Beruhigung.

In einer akuten gesundheitlichen Bedrohung ist von einer emotionalen Stimmungslage auszugehen, die von Angst, Wut, Verstörung und Unsicherheit geprägt ist [10, 24]. Es besteht die Gefahr, dass die Bevölkerung oder manche Bevölkerungsgruppen die Risiken stark überschätzen, was Ängste verstärken und Überreaktionen hervorrufen kann. Die Kommunikation muss dieser Situation Rechnung tragen und darauf abzielen, irrationale Ängste zu verhindern und die Öffentlichkeit – in einem vertretbaren Maße – zu beruhigen. Die „Angst vor der Angst“, d. h. die Sorge vor einer öffentlichen Panik, darf hingegen keine Rechtfertigung dafür sein, Ängste unbegründet zu zerstreuen und Bedrohungen kleinzureden. Risiken müssen klar benannt werden, um Verhaltensempfehlungen rechtfertigen zu können und um glaubwürdig zu bleiben. Sandman fasst diese Schwierigkeit prägnant zusammen: „We have these two very different activities, both called risk communication: alerting people and reassuring them“ [29].

#### Verständnis unterschiedlicher Bedarfe und Partizipation*.*

Die Risikokommunikation muss auf die spezifischen Bedarfe unterschiedlicher Gruppen zugeschnitten sein. Um komplexe kulturelle und sozioökonomische Unterschiede sowie Unterschiede in der Fähigkeit, Risikobotschaften zu verstehen und einzuordnen, zu berücksichtigen, ist es sinnvoll, Betroffene, Bevölkerungsvertretungen und Multiplikatoren in die Formulierung von Botschaften einzubinden [30]. Vulnerable Gruppen fühlen sich oftmals bei Maßnahmen des Risikomanagements nicht ausreichend berücksichtigt [31]. Daher kann es hilfreich sein, die Risikokommunikation bewusst als Dialog bzw. zweiseitige Kommunikation anzulegen [12, 13] sowie die Wirkung der Botschaften kontinuierlich zu evaluieren. Zudem müssen Public-Health-Institutionen mit Gemeinden, Städten, betroffenen Bevölkerungsgruppen und lokalen Organisationen aktiv zusammenarbeiten („community engagement“). Dazu müssen lokale Schlüsselakteurinnen und -akteure an Entscheidungen beteiligt werden, um sicherzustellen, dass Maßnahmen bedarfsgerecht sind und gemeinsam getragen werden [12, 13].

#### Allianzen mit relevanten Akteurinnen und Akteuren*.*

Vernetzungen mit anderen Institutionen und Expertinnen und Experten sollen genutzt werden, um Informationen auszutauschen und die Kommunikation aufeinander abzustimmen. Wenn die Botschaften zwischen verschiedenen Expertinnen und Experten und Akteurinnen und Akteuren nicht konsistent sind, führt das zu Misstrauen in der Bevölkerung [24].

#### Monitoring und Evaluation*.*

Nur wenn man die Zielgruppenerreichung und Wirkung von Risikokommunikation kontinuierlich erfasst und bewertet, können die am besten geeigneten Vorgehensweisen und Tools in einer Public-Health-Notlage identifiziert werden [13]. Eine Evaluation muss verschiedene Fragen beantworten: Inwieweit klärt die gewählte Kommunikationsstrategie Bevölkerungsgruppen über die Gesundheitsrisiken auf? Werden Schutzmaßnahmen verstanden und akzeptiert? Kann Vertrauen bei Kooperationspartnerinnen und -partnern und der Bevölkerung aufgebaut werden [12]? Die Antworten müssen kontinuierlich in die weiteren Planungen zur Risikokommunikation integriert werden. Effektive Risikokommunikation ist ein dynamischer, interaktiver und adaptiver Prozess, denn die Reaktion der Bevölkerung kann sich verändern, z. B. indem Gerüchte entstehen oder Fehlinformationen kursieren [26]. Das Widerlegen von falschen oder verzerrten Informationen gehört daher auch zu einer gelingenden Risikokommunikation.

Die Vorgehensweisen, die sich aufbauend auf diesen Kriterien bewährt haben, werden in Tab. 1 zusammengefasst. Inwieweit sie an spezifische Charakteristika von Public-Health-Krisen angepasst werden müssen, soll im Folgenden am Beispiel der COVID-19-Pandemie aufgezeigt werden.

**Table 1:** 

Kriterium	Vorgehensweise	Besondere Situation/Herausforderungen bei COVID-19
*Glaubwürdigkeit der Botschaften und Vertrauen in Expertinnen und Experten sowie Entscheidungstragende*	Zeitnahe, klare, einheitliche und empathische Kommunikation von Risiken und Maßnahmen	Wissenschaftliche Erkenntnisse sind vorläufig bzw. widersprüchlich; Risiken werden auch in sozialen Medien diskutiert, weitergegeben und teilweise nicht anhand des aktuellen Wissensstands dargestellt; Eindämmungsmaßnahmen führen zu starken persönlichen Einschränkungen und sind teilweise unpopulär. Präventionsparadox: Die Situation ist besser als vorhergesagt, *weil* es drastische Maßnahmen gab
*Eingeständnis von Unsicherheit*	Transparenz darüber, dass Wissen z. T. unsicher und vorläufig ist und sich ändern kann	Der Erreger ist neu; es bestehen Unsicherheiten und fehlende Evidenz zu vielen Themen (Übertragung, Symptome, Langzeitfolgen, Risikofaktoren, Immunität etc.)
*Balance zwischen Alarmierung und Beruhigung*	Klare Benennung von Risiken; Schaffen von Bewusstsein für Risiken; Erkennen und Entkräften von irrationalen Ängsten	Berichterstattung aus stark betroffenen Ländern schürt Ängste und Emotionen; Verlauf scheint schwer kontrollier- und vorhersehbar; bei lang dauernden Einschränkungen droht „Präventionsmüdigkeit“
*Verständnis unterschiedlicher Bedarfe und Partizipation*	Aktive Einbeziehung von Bevölkerungsvertreter/innen in die Strategien und Botschaften; Zuschneiden der Kommunikation auf spezifische Zielgruppen; Einholen von Feedback	Vulnerable Gruppen (z. B. Arbeitsmigrantinnen und -migranten, Obdachlose), die oft schwer erreichbar sind, sind meist höheren Risiken ausgesetzt; Social Distancing/physisches Abstandhalten erschwert partizipative Ansätze; Bedarfe sehr heterogen: Junge Menschen z. B. leiden besonders unter Einschränkungen, haben aber eine vergleichsweise gute Prognose
*Allianzen mit relevanten Akteurinnen und Akteuren*	Vernetzungen mit verschiedenen relevanten Akteurinnen und Akteuren (Wissenschaft, Gesundheitsberufe, NGOs, Medien etc.); Abstimmen von Botschaften	Einheitliche Kommunikation erschwert u. a. durch Föderalismus; Uneinigkeiten in der Wissenschaft und Interessenvertretungen; hohe Arbeitsbelastung einiger relevanter Sektoren
*Monitoring und Evaluation*	Kontinuierliche Erfassung von Zielgruppenerreichung und Wirkung der Risikokommunikation; Erkennen und Entkräften von Gerüchten und Fehlinformationen	Bevölkerung kommuniziert auch in sozialen Medien, was neue, teilweise nicht erprobte oder verfügbare Analysemethoden notwendig macht; öffentliche Einstellungen und Risikowahrnehmungen ändern sich dynamisch; manche besonders betroffene Gruppen sind schwer zu erreichen
*Kommunikation von Zahlen und Grafiken*	Sorgfältige Auswahl von wissenschaftlich untersuchten Formaten und Grafiken, die das Verständnis von Laien erhöhen	Unzählige komplexe Zahlen und Zusammenhänge, die es zu kommunizieren gibt, wie z. B. die Inzidenz, R‑Wert etc.; Unmengen von Grafiken aus dem Datenjournalismus, die nicht auf ihre Verständlichkeit erprobt sind, sondern v. a. ansprechend sein sollen
*Kommunikation des wissenschaftlichen Prozesses*	Der Prozess hinter Einschätzungen und Empfehlungen, v. a. Empfehlungsänderungen sollte kommuniziert werden	Rasante Veränderung des wissenschaftlichen Wissens zu SARS-CoV‑2 und dadurch stetige Veränderungen bei Empfehlungen und Maßnahmen; wissenschaftlicher Diskurs kann für Laien verwirrend sein

## Risikokommunikation in der COVID-19-Pandemie

### Spezifische Herausforderungen an die Risikokommunikation während COVID-19

Seit seinem Ausbruch in Wuhan in der Volksrepublik China Ende 2019 hat sich das neuartige Coronavirus „severe acute respiratory syndrome corona virus 2“ (SARS-CoV-2) rasch auf den Rest der Welt ausgebreitet. Das Virus wird durch direkten Kontakt, Tröpfchen und Aerosole von Mensch zu Mensch verbreitet [32]. Da es noch keine Impfung gegen SARS-CoV-2-Infektionen gibt, bestehen die Schutzmaßnahmen zur Eindämmung von SARS-CoV-2-Infektionen und COVID-19 überwiegend aus nichtpharmazeutischen Maßnahmen in Form von Verhaltensempfehlungen und -regelungen wie Kontaktreduzierung, physischem Abstandhalten, Tragen von Schutz- und Alltagsmasken und Händehygiene [33, 34]. Entsprechendes Verhalten ist besonders wichtig, weil es nicht nur die Einzelne/den Einzelnen vor Schaden schützt, sondern entscheidend dafür ist, die Pandemie als Ganzes in ihren Ausmaßen zu reduzieren.

Risikokommunikation hat bei der COVID-19-Pandemie die Aufgabe, kontinuierlich über die neusten Erkenntnisse zur Übertragung des SARS-CoV-2-Erregers, zu dessen Infektiosität, dem Erkrankungsrisiko und der Prognose von COVID-19 zu berichten. Zum anderen müssen die Ratschläge und Regelungen zum Schutz vor der Infektion kommuniziert werden, die mit umfassenden Einschränkungen des täglichen Lebens und der persönlichen Freiheit einhergehen. Dabei ergeben sich spezifische Herausforderungen für die Risikokommunikation.

In den ersten Monaten der Pandemie gab es Unsicherheiten zu beinahe allen relevanten virologischen bzw. infektionsepidemiologischen Aspekten: die Übertragungswege des Erregers, Krankheitssymptome, Langzeitfolgen der Erkrankung, therapeutische Vorgehensweisen, Risikofaktoren für Morbidität und Mortalität und die Wirksamkeit einzelner Eindämmungsmaßnahmen [35]. Viele dieser Unsicherheiten bestehen noch immer, statt Fakten muss eher von „bester Evidenz zum jetzigen Zeitpunkt“ gesprochen werden [36]. Expertinnen und Experten sowie Politerkerinnen und Politiker müssen wiederholt darauf hinweisen, dass bestimmte Zusammenhänge oder Tatsachen noch nicht verstanden werden, und müssen ihre Meinungen und Empfehlungen immer wieder anpassen oder sogar revidieren [36, 37]. Dies kann die Glaubwürdigkeit der Risikokommunikation beeinträchtigen, muss es aber nicht [37].

Die Ausmaße der Pandemie haben zu einer Flut an offiziellen Mitteilungen, Experteneinschätzungen und Medienberichten geführt. Kommuniziert wird auf verschiedenen Ebenen: von der Wissenschaft, von der Politik und den Behörden, von Journalistinnen und Journalisten und Nachrichtenorganen sowie in großem Umfang über soziale Medien [36, 37], in denen Bürgerinnen und Bürger selbst in ihren Netzwerken die Bedeutung von Risiken und Schutzfaktoren aushandeln [38]. In der COVID-19-Pandemie erweist sich zunehmend als Herausforderung, aus der schieren Menge an Informationen die relevanten und richtigen herauszufiltern [36]. Zudem werden Fehlinformationen und Spekulationen verbreitet und die technischen Fortschritte in der Kommunikation sowie die weitverbreitete Nutzung von sozialen Medien amplifizieren die Auswirkungen von Falschmeldungen und Gerüchten [39]. Man spricht daher auch davon, dass die Pandemie von einer „Infodemie“ (englisch „infodemic“) begleitet wird [37, 40]. Die Reaktion auf diese „Infodemie“ muss Teil der COVID-19-Risikokommunikation sein. Dabei kann sie sich nicht nur auf das Widerlegen von Fehlinformationen und Bekämpfen von Gerüchten beschränken, sondern muss auch proaktiv die digitale Gesundheitskompetenz verbessern, Diskurse in sozialen Medien engmaschig erfassen („social listening“) und den Wissenstransfer zwischen Wissenschaft, Politik und Medien verbessern [36, 40].

Risikokommunikation ist schwierig – sogar unter Bedingungen mit größerer Sicherheit über Übertragungswege oder Wirksamkeit von Maßnahmen. Im Falle einer akuten Public-Health-Notlage, wie sie durch die COVID-19-Pandemie entstanden ist, ist sie angesichts der spezifischen Herausforderungen durch dynamische Entwicklungen, unpopuläre Eindämmungsmaßnahmen und die Flut an Nachrichten über Medien und soziale Onlinenetzwerke noch einmal deutlich erschwert.

### Beispiele der Risikokommunikation zu COVID-19 in verschiedenen Ländern

Einige Autorinnen und Autoren haben sich bereits kritisch mit der Risikokommunikation zu COVID-19 in unterschiedlichen Ländern auseinandergesetzt. Dabei finden sich oftmals ähnliche Muster in den Ausgangslagen und Reaktionen.

Zu Beginn der Pandemie gab es bei Entscheidungsträgerinnen und Entscheidungsträgern in vielen Ländern die ernsthafte Sorge, die drohenden Folgen der Infektionskrankheit könnten die Bevölkerung verstören und in Angst und möglicherweise sogar Panik versetzen, wie beispielsweise Analysen aus Wuhan (China), den USA oder Malaysia zeigen [26, 41–43]. Ziel der Risikokommunikation zu diesem Zeitpunkt war daher, die Öffentlichkeit nicht zu beunruhigen und soziale Stabilität zu erzeugen [44]. Zhang et al. beschreiben, dass man davon ausgehen muss, dass zu diesem Zweck in Wuhan Informationen zum Infektionsgeschehen zurückgehalten worden seien [26]. Auch für die Risikokommunikation in den USA wird beschrieben, dass Public-Health-Expertinnen und -Experten bzw. Politikerinnen und Politiker zu Beginn der Pandemie die Bevölkerung vor allem beruhigt und Risiken heruntergespielt hätten [22, 41, 42]. Die Autorinnen und Autoren der Studien werten diese Strategien als fahrlässig, da sie z. T. auf Kosten eines proaktiven Krisenmanagements gegangen seien, das die beginnende Epidemie möglicherweise hätte eindämmen können. Die zurückhaltende und beschwichtigende Kommunikation der chinesischen Regierung führte laut Zhang et al. zudem dazu, dass sich Gerüchte und Verschwörungstheorien zur neuen Erkrankung v. a. über die sozialen Medien sehr schnell in großem Ausmaß verbreiteten konnten [26], auch als Folge von Misstrauen in die offizielle Kommunikation.

Zur Kommunikation der Eindämmungsmaßnahmen gibt es unterschiedliche Einschätzungen. Ratzan et al. beschreiben, dass die klare, wissenschaftlich begründete und transparente Kommunikation durch Politikerinnen und Politiker z. B. in Deutschland, Taiwan oder Australien dazu geführt habe, Vertrauen in die Regierungen aufzubauen und die Akzeptanz der eingeführten Maßnahmen zu erhöhen [37]. Sandman beschreibt für die USA, dass der sogenannte Lockdown sehr plötzlich, ohne differenzierte Erläuterung und ohne eine echte öffentliche Debatte mitgeteilt und umgesetzt worden sei [41]. Eine Diskussion über die Vor- und Nachteile hätte die Entscheidung transparenter gemacht, argumentiert Sandman. Leask und Hooker hingegen weisen darauf hin, dass es bei der Kommunikation über COVID-19-bedingte Schulschließungen in Australien gerade die öffentliche Debatte war, die zu Verwirrung und Misstrauen geführt habe [45]. Sie heben hervor, dass die inkonsistenten Botschaften unterschiedlicher Entscheidungstragender, z. B. der australischen Regierung, der Territorialregierungen und prominenter Kommentatorinnen und Kommentatoren, zur Abnahme des Vertrauens der australischen Bevölkerung in die offiziellen Empfehlungen und Aktivitäten geführt haben [35, 45].

Die bisherigen Analysen der Risikokommunikation während COVID-19 zeigen, dass die Kriterien für gelingende Risikokommunikation in Public-Health-Notlagen nicht immer leicht umsetzbar sind. Laut Sandman fürchteten sich Expertinnen und Experten sowie Entscheidungsträgerinnen und Entscheidungsträger davor, kritisiert zu werden, wenn sich frühzeitige Warnungen wegen COVID-19 als „falscher Alarm“ herausstellen würden, nachdem schon 2005 eindringlich vor den möglichen schweren Folgen der Vogelgrippe gewarnt worden war, die sich dann – ähnlich wie 2010 die Schweingrippe – als vergleichsweise harmlos herausgestellt hatte [41]. Aufgrund der hohen Bedeutung von Glaubwürdigkeit ist es sinnvoll, die Auswirkungen der Risikokommunikation in Public-Health-Notlagen engmaschig zu evaluieren [36]. In Finnland erfolgt z. B. eine wöchentliche qualitative Auswertung der COVID-19-bezogenen E‑Mails und Social-Media-Botschaften, die an das staatliche Institute for Health and Welfare gerichtet sind. Die Inhalte der Nachrichten werden kategorisiert hinsichtlich des wahrgenommenen Katastrophenpotenzials, der Hypothesen über Infektionsgefahren, des Gefühls der Kontrollierbarkeit der Situation und des Vertrauens in die Regierung. Aus den Ergebnissen werden Empfehlungen für die Risikokommunikation abgeleitet [46].

### Risikokommunikation mit Zahlen und Grafiken

Die COVID-19-Pandemie geht mit einer Flut von Kennzahlen, Statistiken und Grafiken einher, die für die Öffentlichkeit publiziert werden. Sie werden teilweise von der Wissenschaft, häufig jedoch von (Daten‑)Journalistinnen und Journalisten erstellt und verbreitet. Ziel hierbei ist, die Aufmerksamkeit der Leserinnen und Leser zu wecken und zu erhalten, also vor allem ansprechend zu sein. Allerdings werden die Materialien meist nicht darauf getestet, ob sie das Verständnis der Leserinnen und Leser erhöhen oder überhaupt verstanden werden. Viele Menschen, darunter auch Expertinnen und Experten, inklusive Medizinerinnen und Mediziner, tun sich ohne entsprechendes Training schwer, Statistiken und Grafiken zu verstehen [47].

Um dem entgegenzuwirken, bietet die Risikokommunikationsforschung verschiedene Formate, die – gerade auch im medizinischen Kontext – das Verständnis von Statistiken und Zusammenhängen erhöhen [48]. Beispielsweise helfen sogenannte natürliche Häufigkeitsbäume („natural frequency trees“) auch statistisch wenig gebildeten Personen dabei, die Güte von medizinischen Tests zu verstehen [49]. Jüngst wurden sie zur Kommunikation der Tücken von SARS-CoV-2-Antikörpertests vorgeschlagen [50]. Die Vor- und Nachteile von präventiven Interventionen, inklusive Impfungen, können mit sogenannten Faktenboxen dargestellt werden, die Expertinnen und Experten sowie Laien das Verständnis der Inhalte erleichtern [51] und die Bevölkerung dabei unterstützen, informierte Entscheidungen zu Verhaltensweisen zu fällen, die in der COVID-19-Pandemie thematisiert werden. Künftig sollten mehr wissenschaftliche erprobte Kommunikationsformate für Zahlen und Grafiken verwendet werden.

### Kommunikation des wissenschaftlichen Prozesses

Die COVID-19-Pandemie hat die (medizinische) Forschung ins Zentrum der öffentlichen Aufmerksamkeit gerückt. Selbst Boulevardzeitungen diskutieren wissenschaftliche Debatten und Begriffe, und wissenschaftliche Vorabveröffentlichungen (Preprints) werden breit verwendet. Allerdings fehlt vielen in der Bevölkerung das Verständnis des wissenschaftlichen Prozesses und der Wissenschaftskultur. So können Veränderungen von Empfehlungen, die sich aus neuer Evidenz ergeben, die Öffentlichkeit irritieren und verwirren. Außerhalb des Wissenschaftsbetriebs fehlt das Bewusstsein, dass kritische Debatten über wissenschaftliche Erkenntnisse kein Ausdruck von Schwäche der Wissenschaft sind, sondern grundlegendes und notwendiges Element des wissenschaftlichen Fortschritts darstellen. Besonders bei der Kommunikation von Empfehlungen und Empfehlungsänderungen sollten die relevanten wissenschaftlichen Schritte und Prozesse mitkommuniziert werden, um Verständnis hierfür zu fördern.

## Die COVID-19-Pandemie: eine einzigartige Gelegenheit, die Risikokommunikation in Public-Health-Notlagen zu analysieren

Die Wechselwirkungen zwischen Risikokommunikation, Risikowahrnehmung und Verhalten im Kontext der COVID-19-Pandemie sind bislang wenig untersucht worden. Um sich auf künftige Ausbruchssituationen und Public-Health-Krisen besser vorbereiten zu können, sollte die einzigartige Gelegenheit genutzt werden, die die COVID-19-Pandemie für die Analyse der Risikokommunikation bietet. Drei Forschungsprojekte aus Deutschland werden in der Folge vorgestellt.

### COVID-19 Snapshot Monitoring (COSMO)

Um die Risikokommunikation bestmöglich zu gestalten, sollten Informationsstand, das Informationsbedürfnis und die Risikowahrnehmung in der Bevölkerung regelmäßig beobachtet werden [52]. In der COVID-19-Pandemie geschieht dies in Deutschland seit Anfang März 2020 mit einem regelmäßigen Bericht, dem COVID-19 Snapshot Monitoring (COSMO, siehe auch Beitrag von Eitze et al. in diesem Themenheft). Es handelt sich dabei um ein Gemeinschaftsprojekt mehrerer Partnerorganisationen^1^, das sich methodisch an den Empfehlungen der WHO für das Monitoring von COVID-19 orientiert [53].

Wissen, Risikowahrnehmung, Schutzverhalten und Vertrauen in Wissenschaft, Medien und Regierung während des aktuellen COVID-19-Ausbruchsgeschehens sind die maßgeblichen Inhalte des als Querschnitt angelegten Onlinesurveys [54]. Die Studie berücksichtigt dabei, dass eine rein kognitive Wissensvermittlung zur Verhaltensänderung und zur Risikokompetenz nicht ausreichend ist [55] und erfasst auch die emotionalen Aspekte der Risikowahrnehmung wie Ängste, Empörung oder Bedrohungsgefühle im Hinblick auf COVID-19 [56]. Die Studie gibt wichtige Hinweise darauf, ob es Entscheidungsträgerinnen und Entscheidungsträgern bzw. Multiplikatorinnen und Multiplikatoren gelungen ist, klar und vertrauenswürdig zu COVID-19 zu kommunizieren (s. a. Kriterien in Tab. 1).

Einen Teil seiner Fragen setzt das Forschungsteam des Snapshot-Monitorings kontinuierlich ein und ergänzt diesen anlassbezogen entlang aktueller Entwicklungen und gesellschaftlicher Diskussionen. Das Projekt entspricht damit der Forderung, dass Entscheidungsträgerinnen und Entscheidungsträger die unterschiedlichen Bedarfe verschiedener Gruppen erkennen können und regelmäßig erfassen, wie bestimmte Aussagen in der Bevölkerung wahrgenommen und interpretiert werden. Die Ergebnisse sind schnell verfügbar. Das ermöglicht es allen Institutionen, die Risikokommunikation zu SARS-CoV-2/COVID-19 betreiben und Regelungen für Eindämmungsmaßnahmen entwickeln, auf aktuelle Entwicklungen in der Bevölkerung zu reagieren und ihre Risikokommunikation entsprechend anzupassen (wenngleich der Erhebungsmodus – eine reine Onlinebefragung – bei der Interpretation der Ergebnisse berücksichtigt werden muss). Als beispielweise in Deutschland im August 2020 diskutiert wurde, ob es Einschränkungen der Personenanzahl auf Veranstaltungen wie Familienfeiern geben sollte, wurden entsprechende Fragen in die Erhebung aufgenommen. Befragte mit höherem Vertrauen in die Bundesregierung sowie diejenigen, die ein höheres Infektionsrisiko wahrnehmen, beurteilten diese Regelung zu diesem Zeitpunkt eher positiv [57]. Solche und weitere Ergebnisse nutzt das Snapshot-Monitoring auch, um allgemeine Empfehlungen zu publizieren, die konkret für die Risikokommunikation zu SARS-CoV‑2 und COVID-19 genutzt werden können und damit die Infektionsprävention unterstützen.

### Analyse der Entwicklung, Umsetzung und Rezeption von Risikokommunikationsstrategien während COVID-19

Um aus der laufenden Risikokommunikation zu lernen und daraus Lehren für künftige Pandemien zu ziehen, führt das Referat Evidenzbasierte Public Health am Zentrum für Internationalen Gesundheitsschutz des Robert Koch-Instituts die internationale Studie „Risk Communication and Community Engagement (RCCE) during the COVID-19 pandemic: a multi-site international study“ durch. Gemeinsam mit internationalen Partnerinnen und Partnern aus Praxis und Wissenschaft werden die Konzeption, Entwicklung und Umsetzung von Risikokommunikationsstrategien sowie deren Wirksamkeit in 4 Staaten untersucht (Deutschland, Nigeria, Guinea, Singapur). Im Fokus steht auch die Effektivität der jeweiligen Kommunikationsstrategien in der breiten Öffentlichkeit sowie in spezifischen Bevölkerungsgruppen, die Sprach- oder Zugangsbarrieren haben. Die Studie hat eine Laufzeit von Juni 2020 bis Januar 2021.

Die Studie verwendet ein qualitatives Forschungsdesign, das sich auf Kommunikations‑, Sozial‑, Politik- und Anthropologiewissenschaften stützt. Sie umfasst drei Stufen der Datenerhebung: (i) einen Review und eine thematische Dokumentenanalyse, z. B. von Strategiepapieren, Pressemitteilungen und Kommunikationsmaterialien, um einen Überblick über den Umgang der Regierungen mit COVID-19, die eingesetzte Risikokommunikation und die Schlüsselbotschaften in den vier teilnehmenden Ländern zu gewinnen, (ii) halbstrukturierte Interviews mit Schlüsselpersonen, die an der Konzeption und Umsetzung der Risikokommunikation beteiligt waren (z. B. Vertretungen der Regierungen, der Gesundheitsberufe sowie der Medien oder von Nichtregierungsorganisationen), um zu verstehen, wie Risikokommunikationsstrategien entwickelt und umgesetzt werden, und (iii) Fokusgruppendiskussionen und halbstrukturierte Interviews mit Vertreterinnen und Vertretern der wichtigsten Zielgruppen und von Bevölkerungsgruppen, die z. B. aufgrund von Alter, Sprache oder Herkunft Schwierigkeiten haben, Risiken zu verstehen und sich in Diskurse einzubringen. Diese dienen dazu, den Bekanntheitsgrad und die Akzeptanz der Botschaften zu analysieren. Das lässt Rückschlüsse darauf zu, ob klar und vertrauensvoll kommuniziert wurde und die Bedarfe unterschiedlicher Teilpopulationen berücksichtigt wurden.

Die Studie soll Erkenntnisse darüber liefern, inwieweit die Risikokommunikation hinsichtlich COVID-19 die Risikowahrnehmung beeinflusst und zu Schutzverhalten beigetragen hat. Der Vergleich und die Gegenüberstellung von nationalen Strategien, unterschiedlichen Rahmenbedingungen und verschiedenen Zielgruppen können es ermöglichen, erfolgreiche Elemente der Risikokommunikation zu identifizieren und die Risikokommunikation in zukünftigen Gesundheitskrisen entsprechend anzupassen.

### Durchführung von Fokusgruppen mit Jugendlichen zum Umgang mit Informationen während der Coronapandemie

Mit Risikokommunikation kann die für Public-Health-Notlagen relevante Gesundheitskompetenz gefördert werden. In einem qualitativen Forschungsprojekt sollen Erkenntnisse zur Relevanz der Gesundheitskompetenz von Jugendlichen bei der Umsetzung von Infektionsschutzmaßnahmen generiert werden. Einerseits sind junge Menschen zwischen 15 und 34 Jahren die Altersgruppe, bei der in absoluten Zahlen SARS-CoV‑2 am zweithäufigsten nachgewiesen werden kann [58]; zugleich lässt sich im Alltag bei dieser Gruppe ein von den Empfehlungen abweichendes Schutzverhalten beobachten [59, 60], welches in den Medien oft stigmatisierend behandelt wird (Jugendliche als leichtsinnige „Regelbrecher“).

Gleichzeitig berichten Jugendliche, dass COVID-19-bezogene Informationen oftmals nicht für ihre Altersgruppe aufbereitet sind [61]. Sie fühlen sich zudem bei politischen Entscheidungen, die ihre unmittelbare Lebenswelt betreffen, nicht ausreichend gehört, was besonders wichtig ist, da die Eindämmungsmaßnahmen erhebliche Einschnitte in die Alltagsroutinen und das altersentsprechende Verhalten von Jugendlichen mit sich bringen.

Die Studie ist Bestandteil des Projektes „Messung der Gesundheitskompetenz von Jugendlichen (MOHLAA) – Teil 2“ des Robert Koch-Instituts und will explorieren, auf welche Art und Weise Jugendliche welche Informationen zu SARS-CoV‑2, COVID-19 und Infektionsschutzmaßnahmen suchen und erhalten, wie sie diese verstehen und bewerten, erleben und anwenden, ob und wie das Thema innerhalb ihrer Familie und Peergruppe kommuniziert wird. Damit soll das Projekt beitragen zu klären, ob die Risikokommunikation klar, vertrauenswürdig und bedarfsgerecht wahrgenommen wurde.

Das qualitative Studiendesign bietet eine geeignete Gelegenheit, der bisher in der Pandemie wenig gehörten Gruppe der Jugendlichen eine Stimme zu verleihen. Es werden Fokusgruppen eingesetzt, die ein tiefgehendes Verständnis und Nachzeichnen der Wahrnehmung, der Deutung und des Werturteils von Jugendlichen bezüglich der COVID-19-Pandemie ermöglichen können [62, 63]. Damit können die Ergebnisse der Studie zum Verständnis spezifischer Bedarfe in unterschiedlichen Gruppen beitragen und so Risikokommunikation verbessern helfen. Die Studie entspricht damit auch der Empfehlung, Risikokommunikation bewusst als Dialog bzw. zweiseitige Kommunikation anzulegen [12, 13, 30]. Die Studie läuft von Juli 2020 bis Februar 2021.

### Zusammenfassung der vorgestellten Forschungsprojekte

Die dargestellten Projekte analysieren in unterschiedlichen Zielpopulationen (COSMO: Allgemeinbevölkerung, RCCE Study: vulnerable Gruppen, MOHLAA: Jugendliche), wie der Wissensstand zu COVID-19, die Risikowahrnehmung und die Akzeptanz der Eindämmungsmaßnahmen sind. Damit geben sie wichtige Hinweise, ob es gelungen ist, klar, vertrauenswürdig und verständlich zu den COVID-19-Risiken zu kommunizieren. Zudem können in diesen Gruppen besondere Informationsbedarfe erfasst werden, die helfen können, die Risikokommunikation auf Belange bestimmter Zielpopulationen zuzuschneiden. Die Studien entsprechen damit auch der Forderung nach Evaluation und Monitoring der Risikowahrnehmung. Während die COSMO-Berichte eine quantitative Auswertung ermöglichen, erforscht MOHLAA über das qualitative Studiendesign ausführlicher, über welche Kommunikationsformen und -kanäle sich Gesundheitskompetenz zu COVID-19 entwickelt. Die RCCE-Studie verfolgt einen breiteren Ansatz, indem auch die gesamten Risikokommunikationsstrategien von Regierungen analysiert und hinsichtlich ihrer Auswirkungen untersucht werden.

## Fazit

Es gibt bewährte Leitlinien und Goldstandards für effektive Risikokommunikation in Public-Health-Notlagen. Die COVID-19-Pandemie stellt die Risikokommunikation dennoch vor besondere und auch neue Herausforderungen. Dazu gehören vor allem die über Monate ständig wachsende, sich verändernde Evidenz zu Prävention, Verlauf und Therapie der Infektionskrankheit, das Präventionsparadox, komplexe (statistische) Konzepte, die es zu kommunizieren gilt, aber auch die massive öffentliche Kommunikation, die dazu in den sozialen Medien stattfindet. Hinzu kommt, dass im öffentlichen Diskurs die Wissenschaft stark in den Fokus gerückt ist und mit ihr eine Vielzahl an Kennzahlen und Statistiken. Um unter diesen Rahmenbedingungen effektiv kommunizieren und die Risikokommunikation dynamisch anpassen zu können, benötigt man eine kontinuierliche Evaluation der Risikowahrnehmung in Bevölkerungsgruppen; dazu gehört auch das öffentliche Verständnis von numerischen Fakten und Wahrscheinlichkeiten. Mittelfristig können Analysen, die die Wirksamkeit von Risikokommunikationsstrategien untersuchen und vergleichen, helfen, auf zukünftige Krisensituationen effektiv reagieren zu können.

## Footnotes

1 Universität Erfurt, Robert Koch-Institut, Bundeszentrale für gesundheitliche Aufklärung, Leibniz-Zentrum für Psychologische Information und Dokumentation, Science Media Center, Bernhard-Nocht-Institut für Tropenmedizin und Yale Institute for Global Health.
